# Coverage, Timelines, and Determinants of Incomplete Immunization in Bangladesh

**DOI:** 10.3390/tropicalmed3030072

**Published:** 2018-06-25

**Authors:** Nurnabi Sheikh, Marufa Sultana, Nausad Ali, Raisul Akram, Rashidul Alam Mahumud, Muhammad Asaduzzaman, Abdur Razzaque Sarker

**Affiliations:** 1 Health Economics and Financing Research, International Centre for Diarrhoeal Disease Research, Bangladesh (icddr,b), 68, Shaheed Tajuddin Sarani, Dhaka 1212, Bangladesh; nurnabi.sheikh@icddrb.org (N.S.); marufa@icddrb.org (M.S.); nausad.ali@icddrb.org (N.A.); raisul.akram@icddrb.org (R.A.); Rashed.Mahumud@usq.edu.au (R.A.M.); 2 School of Health and Social Development, Deakin University, Melbourne, Burwood, VIC 3125, Australia; 3 School of Commerce, University of Southern Queensland, Toowoomba, QLD 4350, Australia; 4 Laboratory Sciences & Services Division, International Centre for Diarrhoeal Disease Research, Dhaka 1212, Bangladesh; asaduzzaman@icddrb.org; 5 Department of Management Science, University of Strathclyde, Glasgow G4 0QU, UK

**Keywords:** Bangladesh, childhood disease, immunization, timeliness, low vaccination coverage

## Abstract

Immunization has become one of the major contributors to public health globally as it prevents communicable disease, particularly in children. The objective of this study was to estimate the extent of timely immunization coverage and to investigate the determinants of incomplete and untimely vaccination. Methods: The study used data from the latest Bangladesh Demographic Health Survey (BDHS) 2014. A total sample of 1631 children aged 12–23 months who had an Expanded Program on Immunization (EPI) card and immunization history were analyzed. Multivariable logistic regression models were used to determine the significant influencing factors on untimely vaccination (BCG, pentavalent vaccine/OPV, and measles) and incomplete vaccination. The results were presented in terms of adjusted odds ratio (AOR) with a 95% confidence interval and a significance level *p* < 0.05. Results: The proportions of children who received timely vaccinations were 24% for BCG, 46% for pentavalent 3, and 53% for measles, whereas 76%, 51%, and 36% children failed to receive the BCG, pentavalent 3, and measles vaccines, respectively, in a timely manner. The proportion of early vaccination was 3% for pentavalent 3 and 12% for measles. Several significant influencing factors including age, maternal education and working status, awareness of community clinics, socioeconomic status, and geographic variation significantly contributed to untimely and incomplete vaccination of children in Bangladesh. Conclusions: The study identified some key determinants of untimely and incomplete childhood vaccinations in the context of Bangladesh. The findings will contribute to the improvement of age-specific vaccination and support policy makers in taking the necessary control strategies with respect to delayed and early vaccination in Bangladesh.

## 1. Introduction

Immunization has become one of the major contributors to public health globally as it prevents communicable disease, especially among children. The Expanded Program on Immunization (EPI) was established in early 1974 to provide all basic vaccines and to immunize every child around the world [1]. The EPI is a well acknowledged and cost-effective public health intervention that contributes to the reduction of morbidity and mortality worldwide [2,3,4]. Despite the success of EPI, approximately 1.5 million children die each year from vaccine-preventable diseases [5]. Vaccine-preventable diseases remain a potential public health problem in South-East Asia (including Bangladesh) and sub-Saharan Africa because of early or delayed, incomplete, and low vaccination coverage [6]. Bangladesh has had a successful history of immunization and is able to achieve high vaccination coverage against vaccine-preventable diseases. However, the crude vaccination coverage is based on the number of vaccinated children in a specified age cohort (12–23 months) only; it does not indicate the maintenance of scheduled vaccination, though it is also recommended [7]. The negative consequences of early immunization include failure to generate a protective antibody response against vaccine-preventable diseases [8], while delayed immunization takes longer and the child remains susceptible to disease during infancy [9]. It is also evident that the effectiveness of immunization programs tends to be reduced due to delayed vaccination [10]. Further, early received doses are also considered as invalid; though very few children receive early as compared to delayed vaccination [10]. In spite of substantial improvements in maternal and child health and high crude coverage of vaccination, under-five child mortality is still 46 per 1000 live births in Bangladesh [11]. The possible reasons include incomplete vaccination and relatively long delays in timely vaccine administration, which is strongly associated with the increased risk of mortality among children [12]. To maintain the quality of immunization services, the World Health Organization (WHO) recommended improving monitoring and surveillance systems involving age-appropriate vaccinations in low-and-middle income countries (LMICs), including Bangladesh [5]. Therefore, it is necessary to prioritize and monitor the immunization program efficiently in order to reduce delayed and incomplete vaccination and ensure the benefits of immunization. 

Though it is crucial for public health aspects in Bangladesh, limited studies are available to generate evidence about the socio-demographic factors associated with incomplete and untimely vaccination. Furthermore, the available studies focus on specified geographical administrative settings rather than the nation-wide scale [13,14]. However, a few studies have been conducted in neighboring countries [15,16,17], and thus our study may generate evidence that could also be useful for comparison with other settings and to address the knowledge gap. This study utilized the nationwide Demographic and Health Survey (DHS) dataset which provides reliable information on individual-level immunization coverage, timelines, and incomplete vaccination as well as a range of factors that might influence immunization practices. The objective of this study was to estimate the extent of timely immunization coverage and to investigate the determinants of incomplete and failure of timely vaccination. 

## 2. Materials and Methods

### 2.1. Data Source

The study used country representative data from the latest Bangladesh Demographic Health Survey (BDHS), 2014. The survey was designed to provide up-to-date information on socio-demographic, maternal, and child health indicators, including individual level vaccination coverage and timeliness for better future planning and interventions [11]. Childhood vaccination information was collected for all surviving children over the last five-year period. Immunization data were collected based on availability of child health card and maternal recall in those cases when the mother was not able to show child health card or immunization history was not available in the card [11]. Written consent was obtained from the respondents during the interviews. The DHS dataset is one of the largest publicly-available datasets; a mailed consent was taken from the DHS website before conducting this study.

### 2.2. Study Design and Data Collection

A two-stage cluster sampling technique was designed to cover the entire population residing in non-institutional dwelling units in Bangladesh, and the BDHS 2014 was based on a cross-sectional study design. A complete list of enumeration areas (EAs) was used as a sampling framework for BDHS 2014 to cover the whole country, as provided by the Bangladesh Bureau of Statistics (BBS) [11]. In the first stage of sampling, 600 EAs were selected, where 207 were in urban and 393 in rural areas with probability proportional to the EA size. The second stage of sampling involved in selecting 30 households per cluster using a systematic random sampling technique. The survey was implemented from June to November 2014 and data were collected from the selected 17,863 ever-married women aged 15–49 years. A proper sampling weight was used to make the sample more representative of the population at the national level [9,11]. More about the sampling procedure and data collection technique of the BDHS 2014 has been described elsewhere [11].

### 2.3. EPI History and Schedule

Expanded Program on Immunization (EPI) is a priority program of the Government of Bangladesh (GOB) that introduced vaccinations against six preventable diseases (tuberculosis; diphtheria, pertussis, and tetanus; polio; and measles) in 1979. After that, the EPI integrated the hepatitis B (HepB) vaccine, which was primarily initiated in 2003 and was then expanded in 2005 to all districts. The *Haemophilus influenzae* type B (Hib) vaccine was introduced in 2009, and the measles and rubella vaccine in 2012 [11]. Based on the Bangladesh immunization guidelines, children are considered as fully vaccinated when they have received one dose of the vaccine against tuberculosis, Bacille Calmette-Guerin (BCG), three doses of a pentavalent vaccine (DPT, Hib, and HepB), three doses of the polio vaccine (excluding the polio vaccine given at birth), and one dose of the measles and rubella vaccine. If children are not able to receive any one of the recommended doses than they are considered as partially vaccinated [11]. The recommended vaccination schedule for Bangladesh is one dose of BCG at birth or on first contact with health workers, the first dose of penta (penta1) and OPV1 at 6 weeks, penta2 and OPV2 at 10 weeks, penta3 and OPV3 at 14 weeks, and the measles vaccination at 9 months (270 days) of age (Table 1). The intervals between doses (i.e., dose 1 to dose 2 and similarly dose 2 to dose 3) for the pentavalent vaccine and OPV are 4 weeks, respectively, as per the vaccination schedule.

### 2.4. Immunization Coverage and Timeliness

In this analysis, children aged 12–23 months were included to capture the full vaccination coverage and children aged above 23 months were excluded because there was a greater chance of unavailability of EPI cards. Vaccination histories for all vaccines were coded as dummy responses based on whether or not children had received vaccines; in this case we considered the EPI card and also historical recall data when the EPI card was unavailable. Children who were younger than recommended age for each vaccine were excluded from the calculation of immunization coverage. To determine vaccination timeliness, we considered the EPI card of respective children with available vaccination history. Vaccine timeliness was categorized into timely, early, and delayed based on the recommended age of vaccination. Timely vaccination was considered as having received particular vaccines within the recommended age, early vaccination was defined if vaccines were administered prior to the recommended age, and late vaccination was defined if vaccines were administered after the recommended age. However, late and early vaccination were also categorized into three different groups to further explore about early and late vaccinations. Early vaccination categorized as less than 2 weeks early, 2–3 weeks early, and more than 3 weeks early, and, similarly, late vaccination as less than 2 months late, 3–6 months late, and more than 6 months late, respectively. These categories may help better policy formulation to prevent age-appropriate delays. We also performed sensitivity analysis to calculate immunization timeliness.

### 2.5. Explanatory Variables

The selection of the different determinants from the BDHS dataset was based on prior knowledge and published literature. These included age, gender, birth order, birth size, birth seasons, household size, wealth, residence, antenatal care seeking, maternal education and employment status, exposure to electronic media, and geographic location. Birth seasons of the children were categorized as summer (March–June), rainy (July–September), and winter (November–February) based on the seasons of Bangladesh, taking into account the birth place as the home and the corresponding health facility/institution. Birth size was recoded based on mothers’ recollections as normal if the mother perceived an average size or larger, and small/poor if mother perceived a small or very small size of the child. Maternal age was categorized as <20 years, 20–34 years, and >34 years; employment status recoded as ‘employed’ and ‘not employed/housewife’. Drinking water sources were considered as improved (piped into dwelling, piped to yard/plot, public tap/standpipe, tube well or borehole, protected well, rainwater, bottled water), and non-improved (unprotected well, unprotected spring, tanker truck/cart with drum, surface water). Improved toilet facilities (slush/pour flush to piped sewer system, flush/pour flush to septic tank, lush/pour flush to pit latrine, ventilated improved pit (VIP) latrine, pit latrine with slab) and non-improved (facility flush/pour flush not to sewer/septic tank/pit latrine, pit latrine without slab/open pit, hanging toilet/hanging latrine, no facility/bush/field) were also considered. Cooking fuel types were also categorized as clean fuel (electricity, liquefied petroleum gas (LPG), natural gas, and biogas) and polluting fuel (kerosene, coal, lignite, charcoal, wood, straw/grass/shrubs, agricultural crops, and animal dung). Household socio-economic status was measured based on wealth index generated by the composition of selected household assets using principal component analysis (PCA) technique [11].

### 2.6. Analytical Methods

Children aged 12–23 months with an immunization card and EPI history from the BDHS child record dataset were included in this study. However, influential, inconsistent, and missing data were excluded from the analysis. Finally, a total sample of 1631 children aged 12–23 months who had EPI cards and immunization histories were selected and analyzed. Descriptive statistics such as proportion, mean, standard deviation, and frequency distribution were executed to represent the background characteristics of the study participants. Proportions were used to present the immunization coverage and timeliness. Multivariable logistic regression models were used to determine the significant influencing factors for untimely vaccination (BCG, pentavalent vaccine/OPV, and measles) and incomplete vaccination and results were presented in terms of adjusted odds ratio (AOR) with a 95% confidence interval (CI). Before the execution of a multivariable regression model, a bivariate analysis was conducted to trace out the significant factors and statistically significant factors were retained in the regression models. Three separate binary logistic regression models were used to check the effect of different relevant predictors on failure of timely vaccination for the specific vaccines: (1) BCG; (2) pentavalent vaccine/OPV; and (3) measles. All statistical analyses were performed using the statistical software Stata/SE 13.0 and the entire test results were compared with 95% significance level.

## 3. Results

### 3.1. Background Characteristics

Background characteristics of the study participants were presented in Table 2. Almost half of the children were male (52.85%), born at home (59.66%), and lived in rural areas (74.04%), and only 38.27% of them had undergone a confirmed health checkup from a health professional within the two months following birth. The mean maternal age of study children was 24.38 (SD ± 5.53), the proportion of uneducated mothers was 12.65%, 24.29% of mothers were employed, and almost 28% of them were aware about community clinics (CC). Most of the households used polluting fuels for cooking (85.39%), had improved drinking water sources (88.53%) and had hygienic toilet facilities (61.11%). 

**Figure 1:**
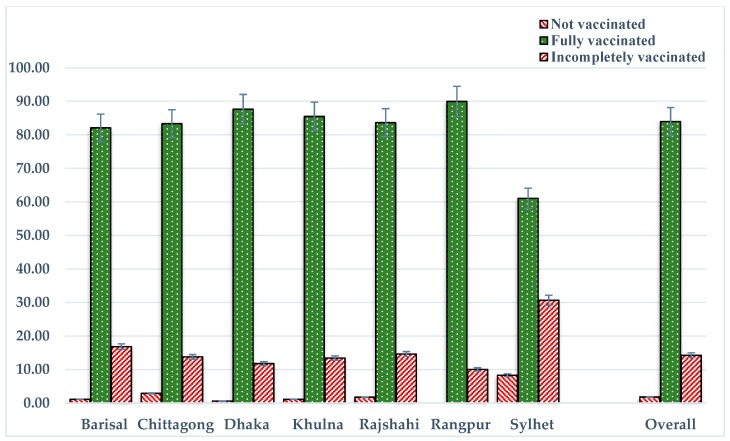
Vaccination status across divisions.

Among all of the study children, 74%, 70%, and 65% had EPI cards with records of BCG, pentavalent 3, and measles vaccinations, respectively (Table 3). The overall vaccination coverage among all study children (including children who did not have EPI cards) was 98% for BCG, 91% for pentavalent 3, and 86% for measles. Similarly, the proportion of fully immunized children was 84%, the proportion of partially immunized children was 14%, and 2% of children had not yet received any vaccine from the EPI schedule (Figure 1). The proportion of children who had received timely vaccination was 24% for BCG, 46% for pentavalent 3, and 53% for measles, whereas 76%, 51%, and 36% of children had delays in receiving the BCG, pentavalent 3, and measles vaccines, respectively (Table 3). The proportion of children who had received early vaccination was 3% for pentavalent 3 and 12% for measles.

The proportions of partially vaccinated (30%) and non-vaccinated (8%) children were higher in the Sylhet division, while full vaccination coverage was higher in the Rangpur division (90%) including all recommended vaccines. However, smaller clustering frequencies for early and delay vaccination are presented in Table 3. In most cases, early vaccination occurred more than 4 weeks early; similarly, delayed vaccination tended to occur more than three months late, with some exceptions.

### 3.2. Failure of Timely Vaccinations and Associated Factors

A number of factors were associated with the failure of the BCG, pentavalent vaccine/OPV, and measles vaccinations. Birth seasons, maternal employment status, source of drinking water, types of toilets, and administrative divisions play a significant role in the failure of timely BCG vaccination (Table 4). The determining factors for the failure of timely pentavalent/OPV vaccinations are birth place, health professional checkup, number of children in the household, maternal educational status, maternal awareness of community clinics, and administrative divisions. In the case of failure of measles vaccination, birth season and birth order, maternal educational status, wealth quintiles and administrative divisions were significantly associated. The study found that the birth season of children was significantly related to the failure to receive BCG and measles vaccines in a timely manner. Children who were born in summer season were 1.53 and 1.49 times more likely to fail to receive the BCG and measles vaccinations, respectively, in a timely manner. The likelihood of failing to receive a timely measles vaccine was 3.11 times higher for those of higher birth order (>5) as compared those of lower birth order (2–3), respectively. In addition, place of birth and healthcare consultation by professionals were significantly associated with the failure to receive timely pentavalent vaccine and OPV vaccinations. Consequently, the children who were born at home and received healthcare checkup by professionals were 2.13 and 1.77 times more likely to fail to receive timely pentavalent/OPV vaccines, respectively. Our results demonstrated that the number of children in a particular household acted as an influencing factor for the failure of timely multi-dose vaccines (pentavalent vaccine/OPV), however, such failures were not observed in single-dose vaccines such as BCG and measles. Maternal education was significantly associated with the failure of timely vaccination. Children of mothers who had no formal education, had completed a primary level of education, and had completed a secondary level of education were 2.34, 2.37, and 2.15 times more likely to have failed to receive multi-dose vaccines as compared with children of mothers who had completed a higher level of education. Similarly, children of unemployed mothers were significantly more likely to be at higher risk of failing to receive the BCG and measles vaccines, respectively (AOR = 1.38, 95% CI = 1.02, 1.93 for BCG and AOR = 1.46, 95% CI = 1.06, 2.00 for measles). However, such relationship was not observed in scenarios of pentavalent/OPV vaccines. 

Maternal awareness is another critical issue for utilization of immunization services. Children whose mothers were not aware about community clinics were significantly (1.40 times) more likely to have failed to receive multi-dose vaccines (AOR = 1.40, 95% CI = 1.06, 1.86). Household characteristics such as source of drinking water and toilet facility were other factors those were significantly associated with the failure of timely BCG immunization. Children from households with poorer accessibility to improved drinking water and hygienic sanitation facilities were at higher risk of failing to receive a timely BCG vaccine. Household size was another determining factor of failure of timely immunizations. Children from households of smaller size (≤5 members) were 1.45 times more likely not to receive the recommended BCG vaccine schedule (AOR = 1.45, 95% CI = 1.06, 1.97 and *p* < 0.05) as compared to larger households. The socio-economic status of the household had a significant impact on vaccination timeliness. The likelihood of noncompliance with the immunization schedule for the measles vaccine was higher among children from the poorest, poorer, and middle-class households as compared to children from the richest households. Living in the Sylhet division was strongly associated with a higher risk of incompliance with the vaccine schedule as compared with the children who were living in the Rangpur division. The odds of failing to receive timely BCG and pentavalent/OPV vaccines were 7.63 and 3.15 times higher among the children who lived in the Sylhet division, respectively, and children of the Dhaka division had a 1.84 times higher risk of failing to receive a timely measles vaccine, as compared to children of the Rangpur division (AOR = 1.84, 95% CI = 1.17, 2.91 and *p* < 0.05).

### 3.3. Factors of Incomplete Vaccinations

In this study, we also tried to trace the influencing factors of incomplete vaccination in Bangladesh. Season of birth, birth order, maternal age and educational qualifications, employment status, hygienic toilet facilities, socio-economic status, and administrative divisions were found as significant predictors of incomplete immunization. Children who were born in the summer and rainy seasons were 1.70 and 2.14 times more likely to be incompletely vaccinated as compared to winter season, respectively. Children whose birth order was 4–5 had 2.10 times more risk of incomplete vaccination. Comparatively, children of the younger mothers (<20, and 20–34 years) were at (3.21 and 3.01 times) higher risk of incomplete vaccination than those of older mothers (>34 years). Similarly, children of less educated mothers (primary level) were at increased likelihood of incomplete vaccination (AOR = 2.72, 95% CI = 1.20, 6.16, *p* < 0.05) than those of mothers with a higher level of education. However, maternal employment also raises the likelihood of incomplete vaccination and children of unemployed mothers were 0.35 times less likely to have been incompletely vaccinated. Children of households with unhygienic toilet facilities were at 89% greater risk of incomplete vaccination (AOR = 1.89, 95% CI = 1.32, 2.71 and *p* < 0.001) than their counterparts. Moreover, lower household socio-economic status was related to higher likelihood of incomplete vaccination than higher socio-economic status. Children from the poorest community had 2.20 times greater risk of incomplete vaccination than the children from the richest community. Similarly, children from Sylhet division were 3.76 times more likely to have incomplete vaccination compared with children from Rangpur division.

## 4. Discussion

Immunization is one of the most effective public health interventions for lowering the burden of disease among young children and averting millions of deaths globally. However, inadequate and incomplete immunization is a significant public health problem in resource-poor countries like Bangladesh. When a certain portion of children receive incomplete vaccinations and/or fail to be vaccinated in a timely manner, there is a possibility of propagating the transmission of the diseases in society. This study put forward the determining factors of incomplete and failure of timely childhood vaccination and contributes to the documentation of pattern of routine immunization uptake in Bangladesh. This study identified several significant influencing factors, including age, education, and working status of mothers, awareness of community clinic, wealth status, and geographic variation that contribute to untimely vaccination and incomplete vaccination of children in Bangladesh.

Our study demonstrated that several factors were significantly associated with untimely vaccination. Factors such as maternal unemployment and lower socioeconomic status (particularly in households with no proper hygienic sanitation systems or potable drinking water) were significantly positively associated with the failure of timely BCG vaccination. Among the determining factors, we found that children of unemployed mothers failed to receive timely vaccinations for BCG/measles. This finding was contradictory the findings of other settings [18]. One reason may be that in Bangladesh most unemployed women are fully engaged with domestic and other non-paid work, and hence they tend to forget their children’s vaccination timing. However, those women are also not financially empowered, which might be another reason for not coming to the vaccination site on time. The study found that those children who lived in lower socioeconomic strata failed to utilize the immunization service in time and could not follow the vaccination schedules, although the timely BCG immunization reduced mortality substantially in Bangladesh [19]. Seasons appeared as another potential influencing factor, although the reason for this difference is not immediately known; future qualitative research will be insightful. It is also noticeable that the children who were born in summer season were less likely to receive the BCG and measles vaccinations in time.

Our study found that children who were born at home were more likely to fail to receive the pentavalent/OPV vaccines. This is supported by previous findings, whereby children born in health facilities had more advantages as compared to those born in households [20]. We found that maternal education is a crucial factor for childhood vaccination, which is in a similar line with other studies in that caregiver education had a positive influence on BCG and measles vaccine coverage [20,21]. As in earlier studies, it is also noted that information barriers such as lack of awareness about community clinics and their activities among mothers increases likelihood of not following vaccination schedules and also the likelihood of incomplete immunization for their younger children [16]. Birth order is also a determining factor of incomplete vaccination and in the case of the measles vaccination we observed that later-born children had a higher risk of failure of timely vaccination; similar findings were also observed in other countries [22].

Our study demonstrated that maternal education and age are significantly associated with incomplete vaccination. Children of younger and less educated mothers were more likely to have been incompletely vaccinated. Globally, similar patterns have been observed; mothers with a lower educational level were less likely to fully utilize immunization services [23,24,25,26]. However, we found for employed mothers, children were at greater risk of incomplete vaccination than those of unemployed mothers. Again, lack of awareness about community clinics, unhygienic toilet facilities, and lower socio-economic status are key factors related to incomplete vaccination. Therefore, the policymaker should be dedicated to investing more resources to increase public awareness and motivation for the timely use of immunization services for children. Those of lower socio-economic strata tend to be deprived of the benefits of vaccination, either due to a lack of awareness or financial isssues i.e., time or resource constraints to access nearby facilities for immunization [16]. It is already well established that when households experience a shortage of food and resources, participation in immunization practices becomes of lower priority [17]. As in an earlier study, we identified geographic barriers as another influencing factor, both for timely use of immunization services and for complete vaccination [16]. According to the administrative regions of the country, the children who lived in Sylhet division were less likely to receive timely vaccines and more likely to have been incompletely vaccinated. The Sylhet division mostly covers a remote hilly and riverine area, and the communication system is more fragile than other regions of the country. However, we did not capture the factors related to supply, such as announcement of campaigns, resources for vaccinations, longer waiting periods, and distances to vaccination sites in this administrative division, although an earlier study found that the information barrier is one of the prime reasons for incomplete vaccination, with some geographic variations [21]. Therefore, proper announcement and precautionary interventions should be encouraged to prevent incomplete vaccination and to explain the positive effects of timely vaccination so that vaccination coverage will be improved.

The study has several limitations for interpretation of results. The study is based on secondary data and the information and the status of child immunization based on either immunization cards or the self-reports of women. Therefore, the potential effect of recall bias on our results cannot be ignored. Therefore, the completeness of vaccination might be underestimated or overestimated. Further, supply side factors were not considered in the study. However, the study results can be generalized at the country level because the study utilized data from the latest nationally representative household survey. Thus, our findings are still significant and relevant in drawing attention to the often neglected aspect of untimely and/or incomplete vaccinations. Hence, a longitudinal study is suggested to explore the factors associated with untimely and incomplete immunization for each type of vaccination.

## 5. Conclusions

The study identified some of the key determinants of untimely and incomplete childhood vaccination in Bangladesh. These findings will contribute to the improvement of age-specific vaccination and support policy makers to develop the necessary control strategies with respect to delayed and early vaccination in Bangladesh. Targeted interventions should be urgently undertaken in order to increase the immunization rates and optimize vaccine effectiveness. These interventions need to focus on those of low socio-economic and educational status in order to improve knowledge on vaccination timing. 

## Figures and Tables

**Table 1:** The Expanded Program on Immunizations (EPI) schedule and timeliness in Bangladesh. Hib: *Haemophilus influenzae* type B.

Diseases	Vaccine	Recommended Age Period	Timely	Early	Late
Childhood tuberculosis (TB)/poliomyelitis	Bacille Calmette-Guerin (BCG)	At birth/0 day	0–28 days	-	>28 days
Diphtheria/tetanus/pertussis/Hepatitis B/Hib pneumonia and meningitis	Pentavalent 1	42 days	39–70 days	<39 days	>70 days
	Pentavalent 2	70 days	67–98 days	<67 days	>98 days
	Pentavalent 3	98 days	95–126 days	<95 days	>126 days
Poliomyelitis	Oral polio vaccine (OPV) 1	42 days	39–70 days	<39 days	>70 days
	OPV 2	70 days	67–98 days	<67 days	>98 days
	OPV 3	98 days	95–126 days	<95 days	>126 days
Measles	Measles	273 days	270–301 days	<270 days	>301 days

**Table 2:** Distribution of background characteristics of the study participants (*n* = 1631).

Variables	*n* (%)
Sex of child	
Male	862 (52.85)
Female	769 (47.15)
Birth year	
2012	460 (28.21)
2013	1171 (71.79)
Birth season	
Summer	537 (32.93)
Rainy	563 (34.52)
Winter	531 (32.55)
Birth order	
1	657 (40.27)
2–3	767 (47.07)
4–5	151 (9.27)
>5	55 (3.39)
Place of birth	
Home	973 (59.66)
Institution	658 (40.34)
Birth size	
Normal	1305 (80.04)
Small/poor	325 (19.96)
Checkup of the infant from a professional	
Yes	624 (38.27)
No	1006 (61.73)
Mother’s number of living children	
1–2	1227 (75.26)
>2	403 (24.74)
Mother’s age (in years)	
Less than 20	326 (20.00)
20–34	1227 (75.23)
35+	78 (4.77)
Mean age (mean ± S.D.)	24.38 ± 5.53
Mother’s education level	
No education	206 (12.65)
Primary	459 (28.14)
Secondary	811 (49.72)
Higher	155 (9.50)
Mother’s employment status	
Not employed	1234 (75.71)
Employed	396 (24.29)
Mother’s awareness of the community clinic	
No	1174 (71.97)
Yes	457 (28.03)
Mother’s access to electronic media	
Yes	663 (40.66)
No	968 (59.34)
Household fuel types	
Clean fuel	238 (14.61)
Polluted fuel	1392 (85.39)
Source of drinking water	
Improved	1444 (88.53)
Non-improved	187 (11.47)
Type of toilet	
Improved	996 (61.11)
Non-improved	634 (38.89)
Household size	
≤5 members	855 (52.42)
>5 members	776 (47.58)
Average household size (mean ± S.D.)	6.07 ± 2.81
Wealth index	
Poorest	373 (22.89)
Poorer	292 (17.93)
Middle	323 (19.80)
Richer	336 (20.59)
Richest	307 (18.80)
Residence	
Urban	423 (25.96)
Rural	1207 (74.04)
Divisions	
Barisal	92 (5.63)
Chittagong	349 (21.43)
Dhaka	622 (38.17)
Khulna	129 (7.89)
Rajshahi	163 (10.01)
Rangpur	146 (8.96)
Sylhet	129 (7.91)

**Table 3:** Adherence to the vaccination schedule for recommended vaccines in Bangladesh based on the Demographic and Health Survey (BDHS) 2014.

Vaccine Name	Time of Vaccination ^1^									Had Vaccination Card (%)	Overall Coverage ^2^ (%)
	Early, *n* (%)				Timely *n* (%)	Delayed, *n* (%)					
	<2 Weeks	2–3 Weeks	≥4 Weeks	Total *n* (%)		<2 Months	3–6 Months	≥7 Months	Total *n* (%)		
BCG (*n* = 1201)	-	-	-	-	293 (24.41)	820 (90.40)	80 (8.83)	7 (0.78)	908 (75.59)	73.64	1597 (97.90)
Pentavalent 1 (*n* = 1201)	11 (8.59)	15 (11.79)	100 (79.62)	126 (10.47)	754 (62.75)	207 (64.34)	108 (33.53)	7 (2.14)	322 (26.77)	73.64	1582 (97.00)
Pentavalent 2 (*n* = 1180)	-	2 (2.82)	61 (97.18)	63 (5.30)	639 (54.18)	-	457 (95.61)	21 (4.39)	478 (40.52)	72.35	1556 (95.40)
Pentavalent 3 (*n* = 1141)	-	-	31 (100.00)	31 (2.74)	527 (46.21)	-	500 (85.87)	82 (14.13)	583 (51.05)	69.96	1489 (91.30)
OPV 1 (*n* = 1201)	12 (9.21)	15 (11.88)	99 (78.91)	125 (10.40)	754 (62.79)	206 (64.35)	107 (33.50)	7 (2.15)	322 (26.81)	73.70	1589 (97.40)
OPV 2 (*n* = 1180)	-	2 (2.87)	60 (97.13)	62 (5.22)	639 (54.14)	-	457 (95.68)	21 (4.32)	477 (40.64)	72.35	1558 (95.50)
OPV 3 (*n* = 1141)	-	-	32 (100.00)	32 (2.81)	525 (46.04)	-	499 (85.82)	83 (14.18)	584 (51.16)	69.96	1491 (91.40)
Measles (*n* = 1053)	2 (1.42)	-	122 (98.58)	124 (11.78)	551 (52.33)	-	-	378 (100.00)	378 (35.90)	64.56	1404 (86.10)

^1^ Includes sample size based on availability of vaccination cards and vaccination dates; ^2^ Includes sample size of all children.

**Table 4:** Factors influencing failure of timely vaccination and incomplete vaccination among children aged 12–23 months in Bangladesh. AOR: adjusted odds ratio; CI: confidence interval.

Variables	Failure of Timely Vaccination			Incomplete Vaccination
	BCG	Pentavalent Vaccine/OPV	Measles	
	AOR (95% CI)	AOR (95% CI)	AOR (95% CI)	AOR (95% CI)
Sex of children				
Male (ref)	1.00	1.00	1.00	1.00
Female	0.92 (0.69, 1.23)	1.08 (0.84, 1.39)	1.14 (0.88, 1.47)	0.90 (0.66, 1.23)
Birth year				
2012	1.06 (0.74, 1.50)	1.04 (0.75, 1.43)	1.1 (0.80, 1.53)	1.15 (0.78, 1.69)
2013 (ref)	1.00	1.00	1.00	1.00
Birth season				
Summer	1.53 ** (1.04, 2.26)	0.94 (0.67, 1.31)	1.49 ** (1.05, 2.10)	1.70 ** (1.09, 2.67)
Rainy	1.27 (0.90, 1.79)	1.06 (0.78, 1.45)	1.13 (0.82, 1.55)	2.14 *** (1.45, 3.17)
Winter (ref)	1.00	1.00	1.00	1.00
Birth order				
1	1.04 (0.71, 1.53)	1.16 (0.83, 1.62)	1.44 (0.97, 2.03)	1.1 (0.72, 1.67)
2–3 (ref)	1.00	1.00	1.00	1.00
4–5	0.99 (0.53, 1.87)	1.68 (0.97, 2.91)	1.20 (0.68, 2.13)	2.10 ** (1.09, 4.02)
>5	1.87 (0.70, 5.04)	2.10 (0.90, 4.88)	3.11 ** (1.29, 7.54)	1.80 (0.71, 4.58)
Place of birth				
Home	1.39 (0.93, 2.07)	2.13 ** (1.50, 3.03)	0.91 (0.64, 1.30)	1.25 (0.78, 2.01)
Institution (ref)	1.00	1.00	1.00	1.00
Birth size				
Normal (ref)	1.00	1.00	1.00	1.00
Small/poor	1.30 (0.89, 1.91)	1.06 (0.77, 1.47)	0.83 (0.59, 1.16)	1.21 (0.84, 1.75)
Checkup of the infant from a professional				
Yes	1.40 (0.94, 2.09)	1.77 *** (1.25, 2.51)	1.33 (0.94, 1.89)	0.93 (0.59, 1.48)
No (ref)	1.00	1.00	1.00	1.00
Number of living children				
1–2	0.67 (0.41, 1.08)	1.48 ** (1.02, 2.18)	0.92 (0.60, 1.39)	1.48 (0.88, 2.51)
>2 (ref)	1.00	1.00	1.00	1.00
Mother’s age (in years)				
Less than 20	1.48 (0.65, 3.41)	0.76 (0.36, 1.62)	1.15 (0.54, 2.44)	3.21 ** (1.02, 10.13)
20–34	1.34 (0.67, 2.70)	0.76 (0.40, 1.45)	1.39 (0.74, 2.62)	3.01 ** (1.05, 8.60)
35+ (ref)	1.00	1.00	1.00	1.00
Mother’s education level				
No education	0.83 (0.41, 1.68)	2.34 ** (1.26, 4.35)	0.93 (0.49, 1.76)	2.25 (0.92, 5.54)
Primary	1.19 (0.65, 2.18)	2.37 *** (1.40, 4.01)	1.49 (0.87, 2.55)	2.72 ** (1.20, 6.16)
Secondary	1.16 (0.68, 1.98)	2.15 *** (1.35, 3.42)	1.20 (0.75, 1.93)	1.58 (0.72, 3.46)
Higher (ref)	1.00	1.00	1.00	1.00
Mother’s employment status				
Not employed	1.38 ** (1.02, 1.93)	0.92 (0.68, 1.24)	1.46 ** (1.06, 2.00)	0.65 ** (0.46, 0.92)
Employed (ref)	1.00	1.00	1.00	1.00
Aware of the community clinic				
No	0.78 (0.57, 1.08)	1.40 ** (1.06, 1.86)	0.92 (0.69, 1.22)	1.36 (1.01, 1.96)
Yes (ref)	1.00	1.00	1.00	1.00
Access to electronic media				
Yes	1.14 (0.74, 1.73)	1.26 (0.88, 1.80)	1.41 (0.98, 2.03)	1.09 (0.69, 1.74)
No (ref)	1.00	1.00	1.00	1.00
Source of drinking water				
Improved (ref)	1.00	1.00	1.00	1.00
Non-improved	2.73 ** (1.03, 7.21)	0.66 (0.36, 1.21)	1.13 (0.58, 2.23)	1.46 (0.90, 2.37)
Type of toilet				
Improved (ref)	1.00	1.00	1.00	1.00
Non-improved	1.42 ** (1.01, 2.03)	0.90 (0.66, 1.23)	0.77 (0.56, 1.05)	1.89 *** (1.32, 2.70)
Household size				
≤5 members	1.45 ** (1.06, 1.97)	1.07 (0.81, 1.40)	1.10 (0.83, 1.46)	0.95 (0.67, 1.33)
>5 members (ref)	1.00	1.00	1.00	1.00
Wealth index				
Poorest	0.55 (0.27, 1.10)	1.33 (0.72, 2.43)	1.89 ** (1.02, 3.51)	2.20 ** (1.05, 4.61)
Poorer	0.54 (0.28, 1.04)	1.16 (0.65, 2.06)	2.17 ** (1.21, 3.89)	1.26 (0.61, 2.60)
Middle	0.92 (0.52, 1.64)	1.13 (0.70, 1.84)	1.64 ** (1.01, 2.66)	1.10 (0.57, 2.14)
Richer	1.02 (0.62, 1.67)	0.8 (0.53, 1.21)	1.47 (0.97, 2.23)	1.01 (0.54, 1.84)
Richest (ref)	1.00	1.00	1.00	1.00
Residence				
Urban (ref)	1.00	1.00	1.00	1.00
Rural	1.18 (0.82, 1.71)	0.95 (0.69, 1.31)	1.14 (0.82, 1.59)	0.75 (0.49, 1.15)
Divisions				
Barisal	1.61 (0.86, 3.01)	1.73 (0.91, 3.29)	0.65 (0.33, 1.26)	1.7 (0.75, 3.85)
Chittagong	3.01 *** (1.85, 4.91)	1.29 (0.81, 2.05)	1.24 (0.77, 2.01)	1.5 (0.77, 2.90)
Dhaka	3.78 *** (2.40, 5.95)	0.98 (0.64, 1.52)	1.84 ** (1.17, 2.91)	1.18 (0.63, 2.23)
Khulna	4.74 *** (2.44, 9.19)	1.83 ** (1.02, 3.27)	0.75 (0.41, 1.39)	1.77 (0.81, 3.88)
Rajshahi	4.96 *** (2.73, 9.02)	1.52 (0.89, 2.60)	1.37 (0.79, 2.38)	1.62 (0.78, 3.37)
Rangpur (ref)	1.00	1.00	1.00	1.00
Sylhet	7.63 *** (3.55, 16.38)	3.15 *** (1.6, 6.18)	1.07 (0.56, 2.05)	3.76 *** (1.84, 7.67)

** *p* < 0.05, *** *p* < 0.01.
